# Faecal glucocorticoid metabolite profiles in diademed sifakas increase during seasonal fruit scarcity with interactive effects of age/sex class and habitat degradation

**DOI:** 10.1093/conphys/coz001

**Published:** 2019-02-06

**Authors:** Stacey R Tecot, Mitchell T Irwin, Jean-Luc Raharison

**Affiliations:** 1 School of Anthropology, University of Arizona, Tucson AZ, USA; 2 Laboratory for the Evolutionary Endocrinology of Primates, School of Anthropology, University of Arizona, Tucson AZ, USA; 3 Department of Anthropology, Northern Illinois University, DeKalb IL, USA; 4 NGO Sadabe, Lot AB64bis, Ankadindravola, Ivato Firaisana, 105 Antananarivo, Madagascar; 5 Co-first authors

**Keywords:** Field endocrinology, habitat disturbance, lemur, primate, season, stress

## Abstract

Glucocorticoids are metabolic byproducts of animals’ physiological responses to ecological or social challenges and are thought to represent an adaptive response allowing beneficial responses to short-term challenges. Glucocorticoid metabolites (GCs) can be assayed non-invasively through faeces and therefore can be a useful tool to gauge the health of populations experiencing natural and/or anthropogenic stressors. However, the response of GCs to anthropogenic stressors varies, with both higher and lower GC levels reported. Here, we describe variation in GC secretion within eight diademed sifaka (*Propithecus diadema*) groups across 1 year. These groups span a gradient of anthropogenic habitat disturbance, including groups in continuous forest (‘CONT’) and disturbed fragments (‘FRAG’), and indicators of health suggest that FRAG groups are negatively impacted by habitat disturbance. We monitored phenology, used focal animal follows to quantify diet and collected faeces (*n* = 547) from which we quantified GC content using enzyme immunoassay. All groups showed elevated lean-season GCs, but with a single, brief peak. GCs were inversely correlated with feeding time. No overall effect of habitat (CONT vs. FRAG) was found, but the lean-season peak was significantly higher in CONT groups. There was a significant season*age–sex interaction; adult females had an attenuated lean-season response compared with groupmates. The observed lean-season ‘challenge’ is consistent with previous lemur studies, as well as mammals in general. Low and largely invariable GC levels in FRAG, within the context of observed health and nutritional declines, suggest that FRAG groups employ a strategy whereby the adrenal response to stressors is downregulated. More research is needed to contextualize our observations of GC variation and health on an individual level, both in terms of corroborating evidence for ecological and social stressors, and longer-term quantification of reproductive success and fitness.

## Teaser

We measured faecal glucocorticoid metabolites (GCs) in Critically Endangered diademed sifaka to understand how food availability, nutritional intake and habitat disturbance impact stress physiology. GCs elevated with food scarcity and correlated negatively with feeding time. Attenuated GCs in fragmented forest groups suggest a downregulated response to a chronically poor environment.

## Introduction

As habitat alteration progresses and few environments remain untouched by humans, determining the effects of anthropogenic change on populations and ecosystems is increasingly important for conservation, and to understand processes of selection (Madliger and Love, 2014; Wingfield, 2008). Effects on behaviour and physiology often precede larger-scale demographic impacts (Ellis *et al.*, 2012), and therefore may be indicators of biologically relevant disturbance and harbingers of population declines. Accordingly, identifying key factors that invoke behavioural and physiological responses can help identify threats to wild populations; these data can then be used to model long-term strategies and evolutionary outcomes in response to anthropogenic disturbance, and design species-appropriate management interventions (Wikelski and Cooke, 2006).

Glucocorticoids have been used to measure the physiological response to environmental stressors in several species (reviewed in Beehner and Bergman, 2017). Glucocorticoids are part of the vertebrate stress response system, which evolved to help individuals respond to dynamic environments. The stress response is an extensive and intricate process involving the immune, endocrine and central nervous systems (Chrousos, 1998). Once a stressor is perceived, the hypothalamic-pituitary-adrenal (HPA) axis is stimulated. As part of a cascade of physiological and behavioural adjustments, glucocorticoids (such as cortisol and corticosterone) are secreted from the adrenal cortex into the blood. The stress response is primarily catabolic, mobilizing energy from long-term survival functions toward immediate needs (Sapolsky, 1987). Once the stressor subsides, a negative feedback loop reduces the response, including lowering glucocorticoid secretions. Therefore, glucocorticoids are expected to reflect the intensity of a *response* to a stressor (Hennessy *et al.*, 1979), and have been termed ‘anti-stress hormones’ (Wingfield and Kitaysky, 2002). If this response is prolonged, long-term functions may become chronically inhibited (e.g. anovulation, increased susceptibility to disease); the stress response becomes maladaptive insofar as long-term glucocorticoid elevations impact health and fitness (Sapolsky, 1998; Sapolsky *et al.*, 2000; Selye, 1978).

Such is the logic behind the ‘cort-fitness hypothesis’ (Bonier *et al.*, 2009), which suggests an inverse relationship between glucocorticoid levels and fitness (Pride, 2005; Rakotoniaina *et al.*, 2017), based in part on observations of elevated glucocorticoids in populations experiencing anthropogenic disturbance (e.g. artiodactyls: Stabach *et al.* (2015); passerines: Lucas *et al.* (2006); primates: Balestri *et al.* (2014); Carlitz *et al.* (2016); Chapman *et al.* (2006); Jaimez *et al.* (2012); Martinez-Mota *et al.* (2007); rodents: Kuznetsov *et al.* (2004)). However, two types of empirical data have failed to conform to this hypothesis. First, some animals in disturbed habitat do not secrete more glucocorticoids despite other indications that their health is negatively impacted (contra Dantzer *et al.* (2014); Dickens and Romero (2013)). Some studies yielded no effect of habitat (Rakotoniaina *et al.*, 2016; Rimbach *et al.*, 2013), while others found lower glucocorticoids in disturbed habitat (e.g. artiodactyls: Sayre (1996); caudates: Homan *et al.* (2003); primates: Aronsen *et al.* (2015); Tecot (2008, 2013); proboscids: Munshi-South *et al.* (2008)), and in populations subjected to a chronic stress regime (Cyr and Romero, 2007). While low glucocorticoids can indicate low levels of stress, they may also indicate tolerance, habituation, attenuation or cessation of the adaptive stress response (Herman, 2013; Homan *et al.*, 2003; Mendoza *et al.*, 2000; Romero, 2004). Second, species that secrete more glucocorticoids in disturbed habitat do not consistently suffer fitness costs (Bonier *et al.*, 2009).

Glucocorticoids are thus context-dependent; elevations can reflect both a maladaptive state of distress and a healthy response to a stressor (Mendoza *et al.*, 2000). It is therefore imperative that contextual information (including potential stressors) be collected alongside glucocorticoids (Breuner *et al.*, 2008), as overly simplistic interpretation of glucocorticoid levels can lead to inappropriate conservation recommendations. This is especially important when causal relationships are difficult to determine, as is the case with many endangered species for whom experimentation would be inappropriate. In addition to measuring baseline levels, the responses of individuals in disturbed and undisturbed habitat to known stressors, such as food scarcity (Cavigelli, 1999; Gómez-Espinosa *et al.*, 2014; Muller and Wrangham, 2004), also should be compared; this can help distinguish between healthy, low glucocorticoid levels that fluctuate in response to challenge, and an attenuated response reflecting an inability to cope in the same way (Tecot, 2008, 2013). Finally, when possible, data on fitness measures should be collected (Beehner and Bergman, 2017; Bonier *et al.*, 2009).

Madagascar’s environment has been described as ‘harsh’ (Wright, 1999) because rainfall and food availability are unpredictable (Dewar and Richard, 2007), drought, frost and cyclones are common (Ganzhorn, 1995; Gould *et al.*, 1999), soils and plants have low productivity (Balko and Underwood, 2005; Ganzhorn *et al.*, 1999; Johnson, 2002) and food scarcity is often prolonged (Bollen and Donati, 2005; Wright *et al.*, 2005). While lemurs are ecologically flexible and have evolved behavioural and physiological coping strategies (Donati *et al.*, 2017), their ability to survive and reproduce can be impacted by these environmental conditions (Gould *et al.*, 1999). Additional challenges are posed by extensive forest loss and anthropogenic disturbance (Harper *et al.*, 2007). Disturbed and fragmented habitat shows even lower plant productivity and predictability (Irwin and Raharison, in press; Tecot, 2008), and mounting evidence is documenting impacts on lemur population health, including shifts in morphometrics, hematology and blood chemistry and parasite prevalence (Irwin *et al.*, 2010; Junge *et al.*, 2011; Schwitzer *et al.*, 2010).

The impacts of environmental factors on glucocorticoid levels have not been investigated broadly in lemurs, but responses have been detected in some species. Glucocorticoid levels elevate with drought and cyclones in *Lemur catta* (Fardi *et al.*, 2018), and fruit scarcity in *L. catta* (Cavigelli, 1999; Pride, 2005), *Eulemur rubriventer* (Tecot, 2008, 2013), *Eulemur collaris* (Balestri *et al.*, 2014) and *Microcebus murinus* (Hämäläinen *et al.*, 2015). Additionally, habitat disturbance altered these environmental effects on glucocorticoids in two frugivorous species, but with opposite patterns. Both studies found elevated faecal glucocorticoid metabolites (GCs) during food scarcity. However, higher GCs were found in *E. collaris* in disturbed forest (Balestri *et al.*, 2014), while lower, almost invariable GCs were found in *E. rubriventer* in disturbed forest (Tecot, 2008, 2013). This latter pattern might be observed when animals reduce energy expenditure, and functions requiring energy (such as HPA axis activity) are attenuated (Fokidis *et al.*, 2012). It is also important to remember the potentially confounding effects of social stress; Gabriel *et al.* (2018) found higher GCs in *L. catta* occupying a more resource-rich environment, but where population density was higher and intergroup competition was more intense. More research on the relationship between glucocorticoids and habitat is necessary to understand why species show different patterns of glucocorticoid variation in response to anthropogenic impacts.

In this study, we investigate GC levels in eight diademed sifaka (*Propithecus diadema*) groups over 1 year and spanning a gradient of habitat disturbance. Diademed sifakas are critically endangered (Andriaholinirina *et al.*, 2014), female-dominant, mostly folivorous lemurs (although they seem to track fruit availability and prefer fruit, when available, over leaves; Irwin, 2008a, 2008b; Irwin *et al.* 2015). Previous studies investigating GC levels in lemurs focused on frugivores; although folivores are thought to be more resilient to disturbance than frugivores, GC levels in folivorous primates can vary with the degree of habitat disturbance (Aronsen *et al.*, 2015; Chapman *et al.*, 2006; Martinez-Mota *et al.*, 2007). In this population, habitat disturbance is associated with differences in behaviour and indicators of reduced health (Irwin *et al.*, 2010, 2015) Specifically, we ask: (1) is there a lean season increase in GCs, presumably reflecting nutritional challenge and does this correlate with decreased availability of fruits or other resources, (2) do groups in more disturbed habitat exhibit higher GCs, (3) do groups in more disturbed habitat experience a different magnitude of the lean-season increase, (4) do different age/sex classes experience a different magnitude of the lean-season increase and (5) does GC level vary inversely with feeding effort and/or degree of frugivory?

## Methods

### Study site

Tsinjoarivo Forest is located 80 km SSE of Antananarivo, atop Madagascar’s eastern escarpment. This region contains unprotected, central domain mid-altitude rainforest, within the corridor including Ranomafana (150 km SSW) and Andasibe-Mantadia (100 km NE) National Parks. The corridor’s western half has been fragmented and degraded by settlers from the central plateau, while the eastern half is minimally disturbed (details in Irwin, 2006).

Research at Tsinjoarivo has focused on three camps: Vatateza (19°43.25′S, 47°51.41′E, 1396 m), Ankadivory (19°42.98′S, 47°49.29′E, 1345 m) and Mahatsinjo (19°40.94′S, 47°45.46′E, 1590 m). Vatateza and Ankadivory are within continuous forest (‘CONT’) in the central part of the corridor but have interspersed low-density human settlement. Mahatsinjo contains ridge-top forest fragments among higher-density human populations at the corridor’s western edge, has experienced higher levels of anthropogenic extraction and exhibits altered forest structure (reduced tree density, crown volume and basal area per hectare) and lower tree diversity (Irwin and Raharison, in press). Fragments’ exact ages are unknown but continuous human occupation began ~30 years ago. Vatateza and Ankadivory are considered ‘CONT’ sites and Mahatsinjo is considered fragmented forest (‘FRAG’). We base this distinction upon FRAG sites’ more extensive past habitat loss and fragmentation (and, therefore, edge creation), as well as botanical variables reflecting more extensive extraction of large trees (Table 1; following Irwin *et al.*, 2015). Sifakas were subject to low-level blowgun hunting in the past, but this has not been observed in the study area since 2000. No groups practice crop-raiding and both sites experience similar predation regimes and current human disturbance.

**Table 1:** Composition of *Propithecus diadema* study groups at Tsinjoarivo during 2008–09, and corresponding home range characteristics and caloric intakes

Group	# Adult female	# Adult male	# Immatures (1–4 years old)	# Infants born June/July 2008	Female reproductive effort	Home range size (ha)/basal area per hectare^a^	Home range quality index^a^	Energy per metabolic body mass^b^ (kJ · BMkg^−0.762^ · day^−1^)
CONT1	2	1^c^	3: 4-year-old female, 2-year-old male, 1-year-old female	2 (both died in October 2008; presumed infanticide)	BR: lactation^d^	83.2/39.6	32.9	1132
					PB: lactation^d^			
CONT2	1	1	2: 4-year-old male, 2-year-old female	1 (disappeared ~April 2009)	Lactation	76.0/44.7	34.0	1350
CONT4	1	1	4: ~4-year-old male, ~3-year-old female, ~2-year-old male, 1-year-old male	1	Lactation	90.2/35.3	31.8	n/a
CONT5	1	1	2: ~2-year-old male, 1-year-old male	1 (died before data collection)	Lactation^e^, gestation/birth	62.9/42.5	26.8	n/a
FRAG2	1	1	2: 4-year-old male, 2-year-old male	1	Lactation	40.1/17.4	7.0	677
FRAG4	1	1	3: 4/5-year-old male, 2-year-old female, 1-year-old male	1	Lactation, gestation/birth	44.6/22.8	10.2	1084
FRAG5	1	1	0	1	Lactation	28.0/25.5	7.1	n/a
FRAG6	1^f^	1	2: 2-year-old female, 1-year-old male	1	Lactation	44.1/19.9	8.8	n/a

^a^From Irwin & Raharison (in press); home range quality index = (home range size (ha) × basal area (m^2^/ha) of trees >5 cm diameter at breast height)/100.

^b^From Irwin *et al.* (2015).

^c^Resident adult male ‘RAD’ was replaced by adult male ‘BG’ in December 2008/January 2009 (between data collection cycles).

^d^BR (Blue–Radio) and PB (Purple–Blue) are the two adult females; lactation interrupted by infant death for both females.

^e^lactation interrupted by infant death.

^f^Died January 2009.

Rainfall at Vatateza totals 2632 mm, with 1697 mm (64%) falling during the single rainy season (December–March). Rainfall at Mahatsinjo totals 2083 mm, with 1307 mm (63%) falling during the rainy season. Temperature is highest in December–January and lowest in June–August. Previous studies distinguished ‘lean’ (roughly April–September) and ‘abundant’ (roughly October–March) seasons, based on observed lean-season reductions in temperature, rainfall and food availability (Irwin *et al.*, 2015).

### Study subjects

Tsinjoarivo sifakas (*P. diadema*) have been studied since 2002 (Irwin, 2008b; Irwin *et al.*, 2015). They live in small groups (2–10 individuals, excluding infants) containing 1 adult male, 1–2 adult females and ≤7 immatures. Aggression is rare (~0.1 acts/h), but adult females are routinely dominant to adult males (Irwin, 2006). Reproduction is highly seasonal: they mate in December, gestate between December and June/July, give birth in June or July and lactate until January/February.

Tsinjoarivo sifakas are largely folivorous (53% of feeding time on foliage, 24% fruits, 15% flowers, 7% seeds), with an abundant season emphasis on fruits and seeds and a lean-season emphasis on leaves and flowers, largely from the fallback food *Bakerella clavata*, a mistletoe (Irwin, 2008b). FRAG groups have lower dietary diversity than CONT groups and their fruits derive largely from *B. clavata* rather than trees. Macronutrient intakes vary seasonally: abundant season intakes of food and macronutrients in CONT groups are up to four times higher than lean-season intakes. FRAG groups largely lack the tree species that provide preferred abundant season fruits and consequently have ‘lean-season-like’ nutritional intakes year-round (Irwin *et al.*, 2014, 2015). Physiological and behavioural differences suggest compromised health and immunocompetence in FRAG groups: lower white blood cell counts (Irwin *et al.*, 2010) and body mass (Irwin *et al.*, submitted).

**Figure 1:**
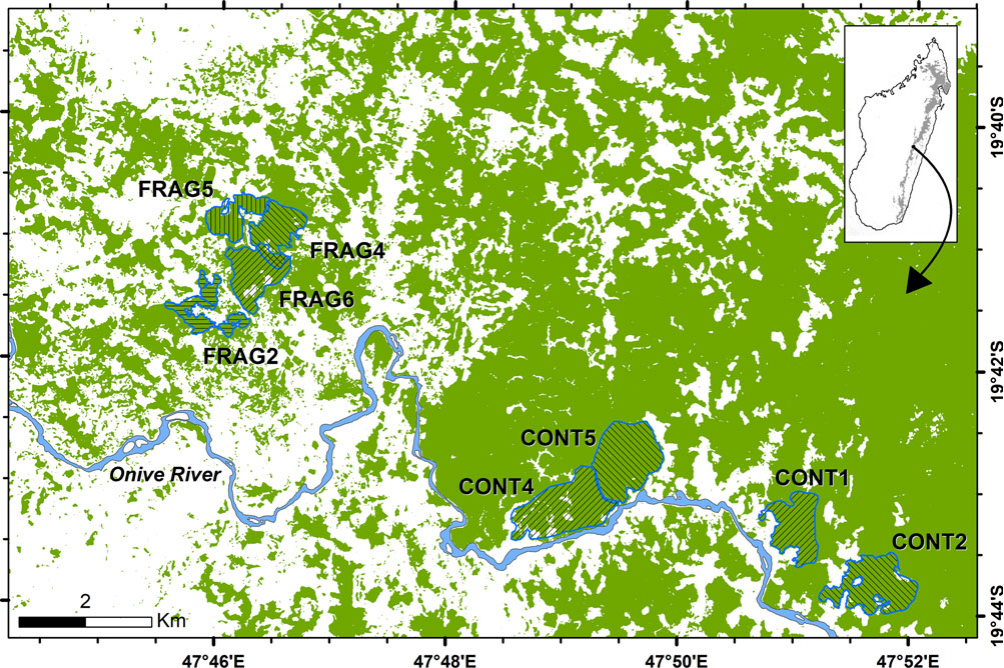
Map of Tsinjoarivo Forest, Madagascar, showing home range of eight *Propithecus diadema* study groups studied during 2008–09.

We studied eight groups (Table 1; Fig. 1): four CONT groups at Vatateza and Ankadivory, and four FRAG groups at Mahatsinjo. All FRAG groups live in isolated fragments: FRAG2 was the sole resident group in a 44.0-ha fragment while FRAG4, FRAG5 and FRAG6 shared a 228.1-ha fragment with another group. All adult females were reproductively active: seven lactated during the first part of the study, while two more lactated during the first part and gestated and gave birth in the second part.

### Data and sample collection

We collected data within 10 ‘data collection cycles’ from 16 September 2008 to 7 September 2009 and aggregated these into five consecutive ‘seasons’ (Table 2); cycles lasted roughly 3 weeks, with the first half being spent at Mahatsinjo and the second half being spent by separate teams (simultaneously) at Ankadivory and Vatateza. These seasons roughly correspond to previous studies (Irwin *et al.*, 2015); seasons 1, 4 and 5 represent the lean season. We accumulated 696 all-day focal animal follows (8–12 h per follow; animals ≥1 year old). Feeding was recorded continuously; for each bout we recorded start and stop time, and plant part and species consumed (bouts were stopped for pauses >10 s).

**Table 2:** Seasonal sampling of faecal glucocorticoid metabolites in *Propithecus diadema* at Tsinjoarivo

Season	Dates	Data collection cycles	# Samples	Reproductive cycle
1 (Lean)	16 September–10 October 2008	1	47	Early Lactation
2 (Abundant)	16 October–17 December 2008	2–4	171	Late Lactation
3 (Abundant)	5 January–4 April 2009	5–7	170	Early Gestation
4 (Lean)	20 April–11 June 2009	8	62	Late Gestation
5 (Lean)	15 June–7 September 2009	9–10	97	Birth
Total			547	

We collected 1179 faecal samples for GC analysis (following Tecot, 2008): we opportunistically collected fresh samples (free from urine contamination) following defecation from identified individuals. Samples (~5 g wet weight) were placed in aluminum foil until the end of the day, when wet weight was recorded. Samples were flattened to increase surface area (following Brockman and Whitten, 1996) and dried within the foil by a campfire until dry (typically 2–3 days). After transport from the field, samples were ground by mortar and pestle and stored in polypropylene sample tubes. Tubes were sealed with parafilm and stored in plastic bags with desiccant.

Concurrent with behavioural data collection, we collected phenology data on 797–819 plants in CONT (average *n* = 431) and FRAG (average *n* = 381) habitats; numbers varied due to plant death and replacement. We aimed to have eight individuals per species per habitat, and the species chosen were roughly equally split to represent abundant species and important species in the sifaka diet (Irwin *et al.*, 2015). The CONT dataset has 87 species and the FRAG dataset has 67 (50 were represented in both). CONT plants were in the home range of CONT1 and CONT2; FRAG plants were in the home range of FRAG2 and the now-dead group FRAG1. Plants were mostly trees but included lianas (3.4%), strangler figs (1.7%) and hemiparasites (2.2%). Plants were monitored monthly for flowers, fruit and young leaves. Each variable was scored on a percentage crown coverage basis ranging from 0% (absent) to 100% (theoretical maximum).

### Faecal glucocorticoid assay

We measured GC metabolites in 547 faecal samples in the Laboratory for the Evolutionary Endocrinology of Primates (University of Arizona). We ensured even sampling by including 1–2 samples from each individual during each week that group was followed. If more than one sample per individual was available per day, we chose the earliest sample. To minimize effects of diel variation, we analyzed only samples collected before noon, except two samples (13:49 and 14:56) for which a week had no morning samples for a given individual. We extracted 0.1 g per sample into an ethanol–water (50:50) solution (Strier and Ziegler, 1997), vortexed each sample for 10 min, centrifuged for 10 min at 3000 rpm, then poured off the 5-ml supernatant into 7-ml borosilicate vials. To free conjugated steroids, we separated 1 ml and added 4 ml ethyl acetate. We vortexed each sample for 8 min and centrifuged for 3 min at 1000 rpm, and then aspirated the solvent into culture tubes. We evaporated samples in a water bath under airflow, and refrigerated each in 1-ml ethanol until assay.

We measured faecal GCs using an in-house competitive binding enzyme immunoassay (EIA), following Ziegler and colleagues (1995), with the modification of standards prepared in alcohol (Sousa and Ziegler, 1998). We used antibody (R4866 polyclonal anti-cortisol anti-serum) and cortisol horseradish peroxidase from C. Munro (UC Davis). We evaporated 100 μl of each sample and diluted them 1:2 in EIA buffer. We added 250 μl enzyme-labeled antigen to the standards and each sample, and added 100 μl of the mixture to each well in the plate for binding during a 2-h incubation. Solid-phase washing separated bound and free GCs. We added 100 μl substrate and calculated absorbance using a Biotek Epoch spectrophotometer. Absorbance was read at 415 nm with a reference filter of 570 nm.

We used tests of parallelism and accuracy to validate the ability of the assay to measure adrenocortical activity (Whitten *et al.*, 1998). No differences were detected between the slopes of serially diluted sample and serially diluted cortisol standard (*F*_9,10_ = 1.264, *P* = 0.29). We determined the accuracy of the assay by adding 100 μl *P. diadema* faecal sample pool to each of the standard curve points in duplicate (137.74 ± 4.80% standard error, *n* = 5). Inter- and intra-assay coefficients of variation for high and low concentration sample pools, respectively, were 7.5 and 12.3% (intra) and 19.4 and 19.3% (inter), *n* = 18. Cross-reactivity was reported as 100% with cortisol (Munro and Stabenfeldt, 1985). GC concentrations are expressed in ng/g faeces.

### Data analysis

To examine covariation between GCs and phenology, we expressed the monthly availability of plant parts (flowers, fruit and young leaves) as the percentage of stems bearing some (i.e. >0%) of the food type. Because peak GCs are often brief, yet biologically quite meaningful, we determined which data collection period had peak GC concentrations, then compared CONT and FRAG GCs using a Wilcoxon Rank-Sum test.

We used linear mixed models (LMMs) to explore variation in GC levels, using the nlme package (Pinheiro *et al.*, 2015) in R (R Core Team, 2015). We used log-transformed GC levels, as these greatly improved normality of residuals (assessed by histograms and Q–Q plots). Because groups and individuals were sampled repeatedly, we initially included both group (*n* = 8) and individual nested within group (*n* = 36) as random effects (intercept) in the model. First, we compared a fully-loaded model (fixed effects plus interactions, with group and individual as random effects) to a model with a simplified random effect structure (group only) using likelihood ratio tests (LRTs; threshold for inclusion *P* < 0.05) in R function ‘anova’ (REML model fitting). We adopted the simplified model if retaining the ‘individual’ term did not significantly improve fit; otherwise, we retained the first model. Following this, we tested fixed factors in the following order: three-way interactions (if present), two-way interactions, then single factors. At each stage, we assessed factors by comparing models with and without the factor, and we retained the factor if the LRT was significant using ‘anova’ (ML model fitting). When we retained any three-way interaction, we did not test two-way interactions and fixed factors; when we retained any two-way interaction, we did not test main effects of fixed factors. We report final results using REML models. All LMMs were tested for multicollinearity (VIFs < 3.0).

In the first two models, we used all faecal samples (*n* = 547). For the first model we examined the effects of site (CONT/FRAG), season (lean/abundant), age/sex class (adult female, others) and all interactions. We chose this simplified age/sex categorization because data inspection (both boxplots and results of exploratory models) revealed similar responses in adult males and immatures. For the second model, we examined the effects of site (CONT/FRAG), monthly phenology scores for flowers, fruits and young leaves (% of stems bearing the item), age/sex class (adult female, others) and two-way interactions between Age/Sex and phenology scores. Phenology scores were habitat-specific (CONT/FRAG) and converted to *z*-scores.

Our third model tested for links between GCs and diet characteristics. Because samples were collected opportunistically within groups (i.e. from focal animals and others), we did not always have feeding data corresponding to samples. Instead, we examined the relationship between individual GC averages within data collection cycles (28 individuals; total *n* = 327) and three feeding variables (average daily feeding time, average daily feeding time devoted to fruit/seed and average percentage of total daily feeding time devoted to fruit/seed during that cycle). We plotted all three relationships, but built a LMM using only the first two variables, because including both the second and the third caused multicollinearity (VIFs between 4.0 and 25.0).

We recognize that we are using separate LMMs to predict GCs based on different predictor variables (defined seasons, food availability and diet characteristics), and that these predictors are correlated with one another. A simpler approach would be to choose a single type of predictor to stand in for the others. However, we prefer a broader, exploratory approach because of the unknown nature, and likely complexity, of the causal relationships. Season may affect GCs either through stressful environmental conditions (temperature and rain), timing of reproductive events and associated energy expenditure and social dynamics, and finally seasonal variation in food availability (which may or may not be reflected in diet characteristics such as degree of frugivory and time spent feeding).

We report LRTs and associated *P*-values for all factors tested; for intercepts and factors retained in models we report coefficient, *t*-values and *P*-values; although interpreting these is more complex than LRTs, they can be useful in comparing models (particularly when a significant interaction precludes LRT testing of fixed factors).

## Results

### Intra-annual variation in food availability and GC levels

**Figure 2:**
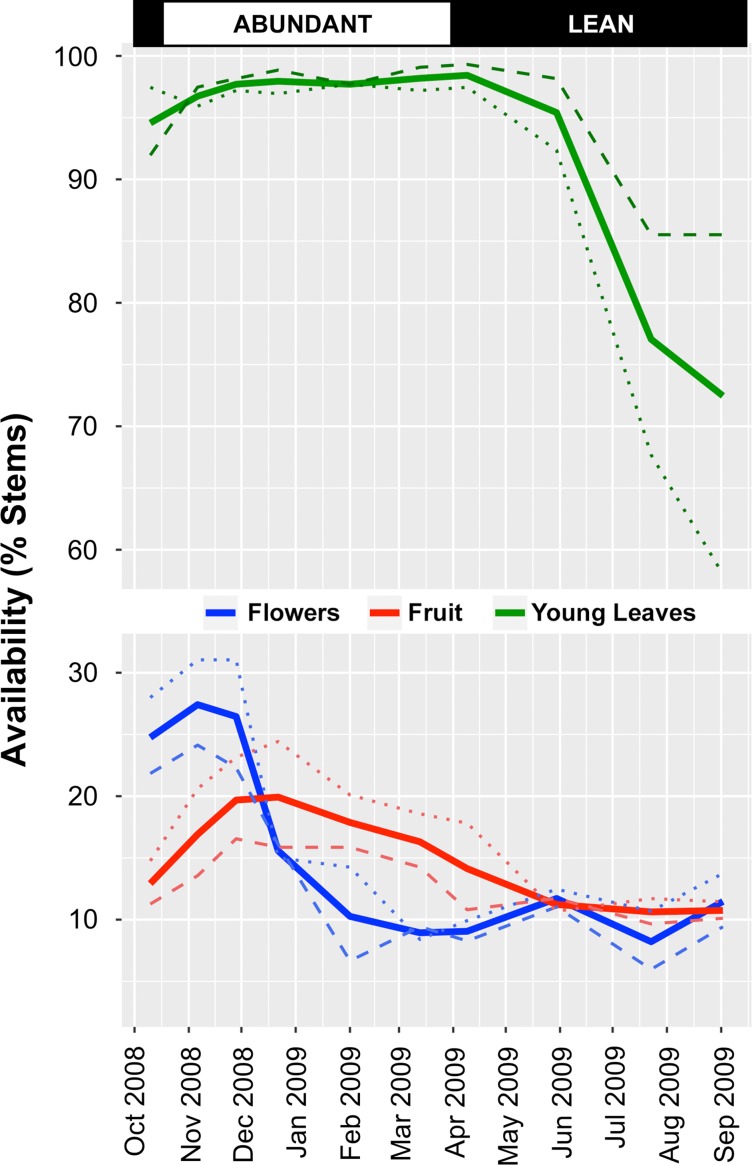
Seasonal variation in production of plant parts relevant to sifakas during 2008–09 at Tsinjoarivo, Madagascar (percentage of stems bearing flowers, fruits and young leaves), showing overall dataset (solid line), as well as CONT habitat (dashed line) and FRAG habitat (dotted line).

**Figure 3:**
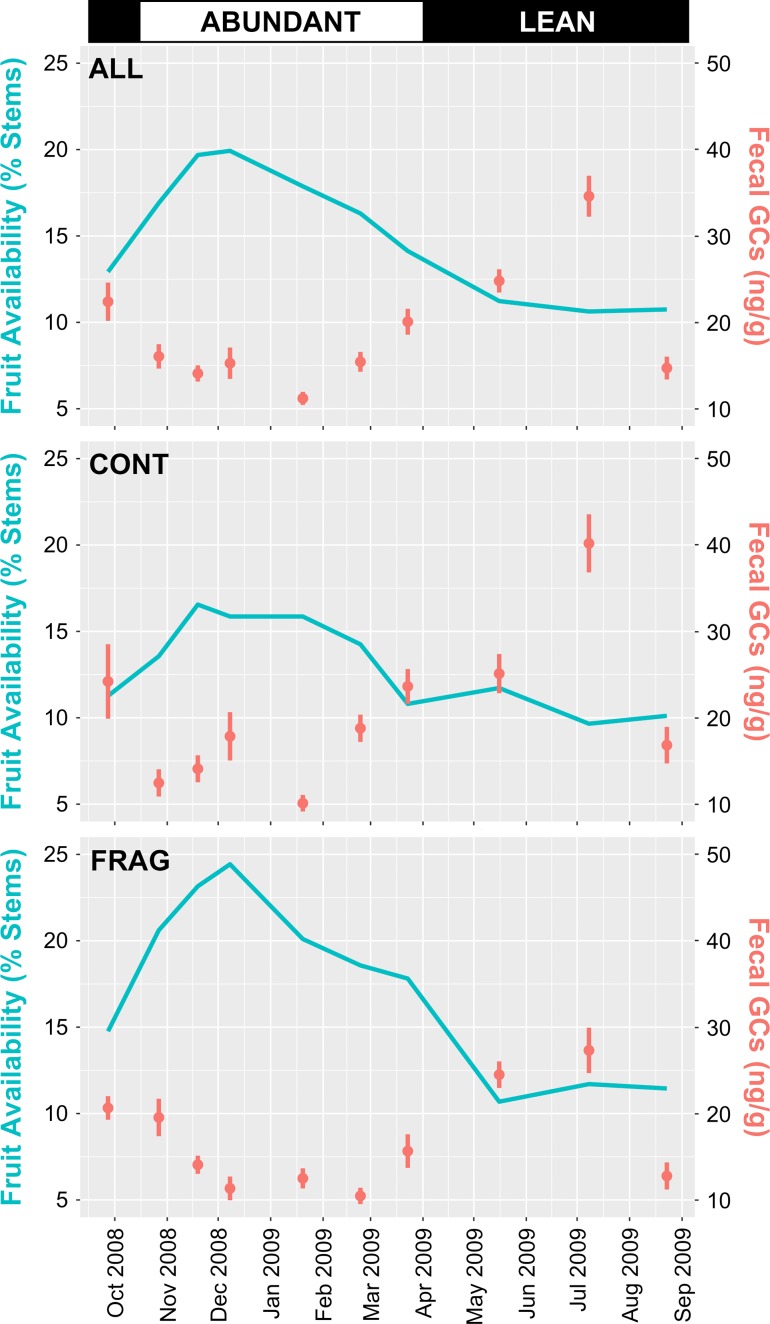
Seasonal variation in fruit availability and faecal glucocorticoid metabolite concentrations within 10 data collection cycles for *Propithecus diadema* at Tsinjoarivo, Madagascar. Cortisol concentrations within each data collection cycle are plotted as mean ± SE.

Phenological data (Fig. 2) were consistent with previous definitions of Tsinjoarivo’s abundant and lean seasons: fruit abundance was above average over data collection cycles 2–6 (mid-October to mid-March, abundant season). This period coincided with lower GC concentrations in the pooled sample, and for CONT and FRAG habitats considered individually (Fig. 3).

CONT and FRAG groups exhibited similar seasonal variation, although at the peak in data collection cycle 9 (15 June–3 August, lean season), CONT groups’ peak (40.2 ± 18.4 SD ng/g, *n* = 30) was 47% higher than FRAG groups (27.3 ± 12.6 SD ng/g, *n* = 23). This difference was significant (Wilcoxon Rank-Sum test, W = 494, *P* = 0.007), and coincides with the lowest recorded fruit availability (10.63% of stems). CONT groups’ GCs were also higher in the other lean-season cycles (1, 8 and 10), though with lower contrast between sites.

The peak seems to be driven by key animals, with concentrations above 45 ng/g seen only in six animals, of mixed age/sex classes, all from CONT groups: CONT1:JUV (89.0 ng/g, *n* = 1, 2-year-old female), CONT1:PG (51.8 ng/g, *n* = 2, 3-year-old male), CONT2:RAD (60.3 ng/g, *n* = 1, adult female), CONT4:AM (47.7 ng/g, *n* = 2, adult male), CONT4:BB (65.1 ng/g, *n* = 2, 3–4-year old male) and CONT4:JUV (52.0 ng/g, *n* = 2, 2-year-old male).

### Effects of season, site and age/sex class

**Figure 4:**
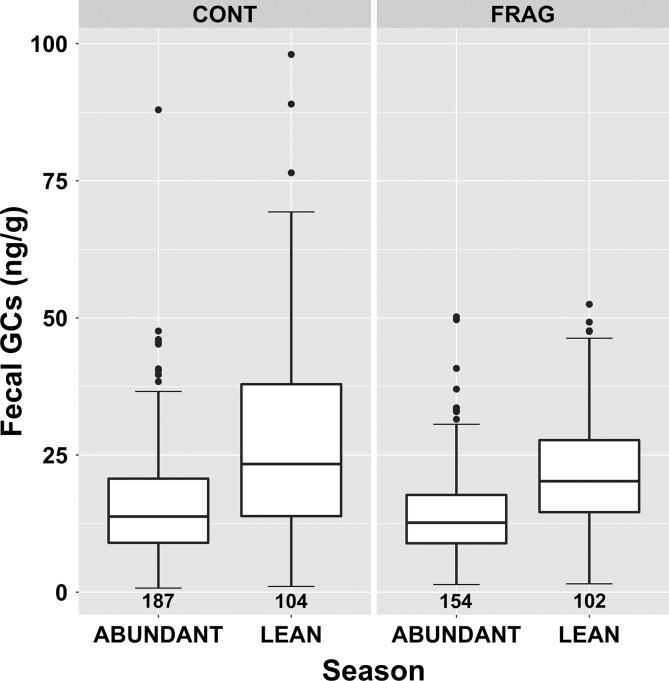
Variation in faecal glucocorticoid metabolite concentration among *Propithecus diadema* individuals at Tsinjoarivo occupying intact (CONT) and degraded/fragmented (FRAG) habitat in the abundant and lean seasons; sample size noted beneath each boxplot.

Average GC concentrations were higher in the lean season for both sites (Fig. 4); the contrast was greater for CONT groups than for FRAG groups. A simple model using only season to predict GCs (not shown) found an increase from 12.48 to 20.06 ng/g between abundant and lean seasons (model predictions). The apparent difference between sites is reflected in predicted means from a model including site, season and the interaction term (CONT: 12.8–21.3 ng/g, +66%; FRAG: 12.2–18.9 ng/g; +55%).

**Figure 5:**
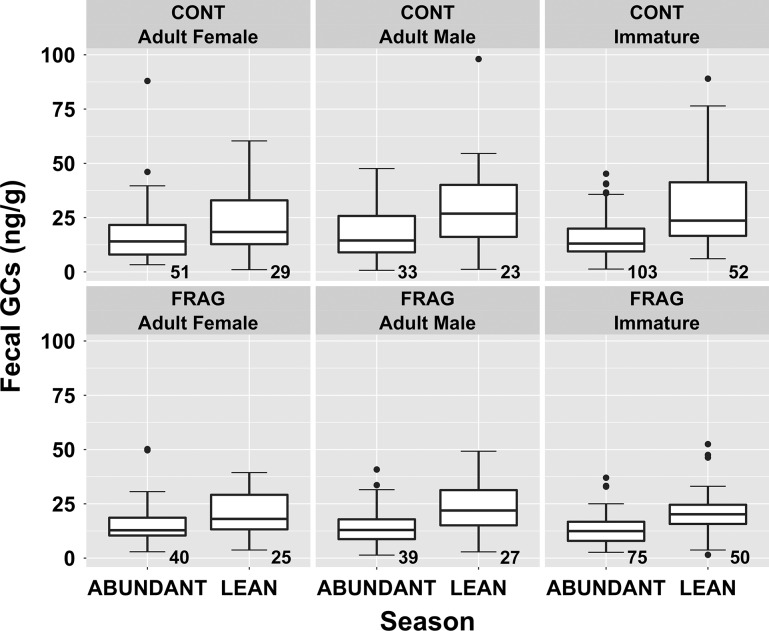
Variation in faecal glucocorticoid metabolite among age/sex classes of *Propithecus diadema* across two habitats in the abundant and lean seasons at Tsinjoarivo; sample size noted beneath each boxplot.

Effects of age/sex class were more subtle: all had similarly low abundant season levels, but adult females experienced lower lean-season elevations (Fig. 5). The apparent difference among age/sex classes is reflected in predicted means from a fully-loaded model including site, season, age/sex (adult female, adult male and immature) and all interactions (not shown). CONT adult females increased 33% (13.3–17.7 ng/g) in the lean season, and FRAG adult females increased 34% (13.5–18.0 ng/g). Adult males increased more (CONT: 13.9–22.1 ng/g, +59%; FRAG: 12.3–19.3 ng/g; +57%), as did immatures (CONT: 12.2–23.1 ng/g, +89%; FRAG: 11.4–19.0 ng/g, +67%).

The model in Table 3 included site, season and age/sex (AF vs. others, to reflect the similarity between adult males’ and immatures’ responses). The Season*Age/Sex interaction was retained; the final model contained this term plus all three fixed factors (though only season had a significant *T*-value). Using this model yields predicted values showing a small lean-season increase for adult females (CONT: 13.8–18.5 ng/g, +34%; FRAG: 12.8–17.1 ng/g; +34%) compared with other animals (CONT: 12.6–21.7 ng/g; +72%; FRAG: 11.7–20.1 ng/g; +72%).

**Table 3:** LMM investigating faecal glucocorticoid metabolite variation in *Propithecus diadema* at Tsinjoarivo using site (CONT vs. FRAG), season (abundant vs. lean) and age/sex class (adult female, others) as predictors (*n* = 547 samples); group was included as a random factor. Bolded text indicates statistical significance (*P* < 0.05)

Final fitted model: site, season, age/sex (AF/other)					
Fixed effect	Coefficient ± SE	*T*	*P*	LRT	*P*
(Intercept)	1.14 ± 0.053	**21.25**	**<0.0001**		
Site FRAG	−0.033 ± 0.066	−0.49	0.6		
Season LEAN	0.126 ± 0.048	**2.59**	**0.010**		
Age/Sex Other	−0.039 ± 0.035	−1.14	0.26		
Site × Season				0.43	0.5
Site × Age/Sex				0.98	0.3
Season LEAN × Age/Sex Other	0.110 ± 0.056	1.95	0.052	**3.92**	**0.048**
Site × Season × Age/Sex				0.17	0.7

The possibility that these results might reflect a simple effect of age seems unlikely: a model using four fixed factors (site as CONT/FRAG, season as abundant/lean, age as adult/immature and sex) yielded no significant interaction terms, and retained only season as a significant predictor of GCs.

A second model in which the dichotomous ‘season’ variable was replaced with phenological scores for flowers, fruit and young leaves (Table 4) showed that fruit availability was a significant negative predictor of GC levels, while young leaf availability was a significant positive predictor of GCs; neither flower availability, nor interactions between age/sex and phenology were retained in the models.

**Table 4:** LMM investigating faecal glucocorticoid metabolite variation in *Propithecus diadema* at Tsinjoarivo using site (CONT vs. FRAG), availability of flowers, fruits and young leaves from phenology data (CONT- and FRAG-specific *z*-scores) and age/sex class (adult female, others) as predictors (*n* = 547 samples); group was included as a random factor. Bolded text indicates statistical significance (*P* < 0.05)

Final fitted model: site, fruit availability, age/sex (AF/other)					
Fixed effect	Coefficient ± SE	*T*	*P*	LRT	*P*
(Intercept)	1.18 ± 0.032	**37.30**	**<0.0001**		
Site FRAG				0.31	0.6
Age/Sex				0.02	0.9
FL Availability				0.75	0.39
Fruit Availability	−0.141 ± 0.016	**−8.61**	**<0.0001**	**67.92**	**<0.0001**
YL Availability	+0.039 ± 0.017	**2.27**	**0.02**	**5.05**	**0.02**
Age/Sex × FL Availability				2.80	0.09
Age/Sex × Fruit Availability				1.55	0.21
Age/Sex × YL Availability				0.18	0.7

### Relationships with feeding variables

**Figure 6:**
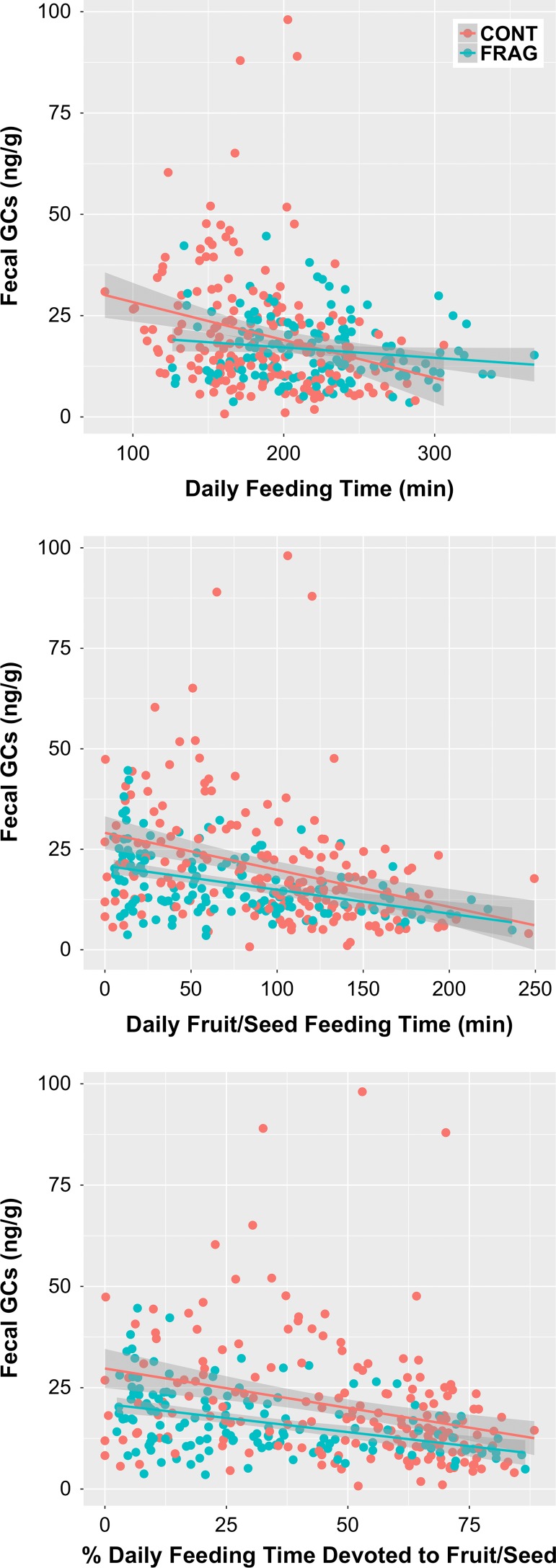
Bivariate plots illustrating the relationship between three key feeding variables and faecal glucocorticoid metabolite concentrations for *Propithecus diadema* at Tsinjoarivo, Madagascar; data points are averages for individuals (*n* = 36) within each of 10 data collection cycles (total *n* = 327).

Individual averages within data collection cycles show that GC levels decrease with daily feeding time, daily fruit/seed feeding time and the percentage of daily feeding time devoted to fruit/seeds (Fig. 6). LMM fitting (Table 5) showed that only average daily fruit/seed feeding time was retained as a significant negative predictor of GC level. Neither of the feeding variables interacted significantly with site, meaning animals in the two habitats responded similarly.

**Table 5:** LMM investigating faecal glucocorticoid metabolite variation using site (CONT vs. FRAG) and two daily feeding variables as predictors (*n* = 327 individual averages within data collection cycles); group was included as a random factor. Bolded text indicates statistical significance (*P* < 0.05)

Final fitted model: site, two feeding variables				LRT	*P*
Fixed effect	Coefficient ± SE	*T*	*P*		
(Intercept)	1.345 ± 0.037	**34.96**	**<0.0001**		
Site FRAG				0.99	0.32
Daily Feeding Time (min)				0.00	1.0
Daily Fruit/Seed Time (min)	−0.0019 ± 0.00025	**−7.67**	**<0.0001**	**35.84**	**<0.0001**
Site:Daily Feeding Time				1.28	0.26
Site:Daily Fruit/Seed Feeding Time				0.24	0.6

Factors were tested stepwise, from bottom to top. An expanded model including Age/Sex (Adult Female/Other) and two-way interactions between Age/Sex and the feeding variables reduced to the same model (data not shown).

## Discussion

Faecal GCs are a useful proxy for HPA axis activation in many wild vertebrates, but one difficulty has been that both healthy and distressed individuals can have low GC levels. Our study yielded a complex pattern of variation in GC output among Tsinjoarivo sifakas, with season, site, and age/sex all seeming to play some role, in addition to interactions between variables. We discuss these findings alongside previous behavioural, ecological and health studies of this sifaka population to help contextualize hormonal profiles and interpret our results.

### Do sifakas experience a lean-season GC response?

Our results confirm previous suggestions that Tsinjoarivo’s lean season (April–September) is challenging for sifakas: it is characterized by lower temperatures and rainfall, and flower and fruit scarcity. Sifakas reduce fruit consumption in favour of leaves and mistletoe flowers, and reduce their ranging, activity levels and feeding time/intakes, possibly because harmful plant secondary metabolites limit intakes (Irwin, 2008a, 2008b). Consequently, CONT groups experience reduced lean-season nutritional intakes, with energy and most nutrients at less than half of abundant season levels (Irwin *et al.*, 2014, 2017); those in disturbed fragments also exhibit reduced intakes, though abundant season intakes were lower to begin with.

Here, we report a strong link between GC production and this seasonal variation: lean-season GCs were higher, and GCs were negatively correlated with fruit production and negatively correlated with leaf production. Finally, GCs are negatively correlated with daily feeding time, daily fruit/seed feeding time and the degree of frugivory. These results suggest that reduced lean-season nutrient intakes (and possibly the physiological challenge of increased plant secondary metabolite intakes) activate the HPA axis, reflecting shifting energetic status and are consistent with previous results that sifakas, despite being considered folivores, actually prefer fruit and depend energetically on this resource (Irwin, 2008b; Irwin *et al.*, 2015). Sifakas may lose weight in the lean season and regain it in the abundant season; this hypothesis could be tested through more frequent capture and/or non-invasive measures of fat catabolism (Vogel *et al.*, 2012).

It is possible that these results reflect the effects of different diets on hormone metabolism and/or excretion (Dantzer *et al.*, 2014), but they are consistent with findings in a diversity of mammals and birds with a variety of diets. For example, this finding is in line with all previous lemur studies, which show lean-season glucocorticoid elevations for at least some individuals or groups (Balestri *et al.*, 2014; Cavigelli, 1999; Gabriel *et al.*, 2018; Pride, 2005), as do monkeys (Behie *et al.*, 2010; Dunn *et al.*, 2013; Foerster and Monfort, 2010; Sapolsky, 1986) and apes (Muller and Wrangham, 2004). We defined the lean season based on a combination of reduced food availability, temperature and rainfall (Irwin *et al.*, 2015). Seasonal glucocorticoid fluctuations can also occur in response to climate, such as rainfall (Carnegie *et al.*, 2011; Gesquiere *et al.*, 2008; Tecot, 2008; but see Rangel-Negrin *et al.*, 2009) and temperature (Beehner and McCann, 2008; Charpentier *et al.*, 2018; Weingrill *et al.*, 2004), likely reflecting shifting metabolic demands associated with food availability as well as thermoregulation. Though we cannot isolate the effects of climate in our analysis, both phenology and observed variation in nutrient intakes (Irwin *et al.*, 2015) corroborate the idea that seasonal food scarcity drives GC variation in sifakas.

In terms of the interaction between reproductive and habitat seasonality, the lean-season GC peak (15 June–3 August) corresponds to the birthing season. Glucocorticoids increase throughout gestation in humans (Lockwood *et al.*, 1996), monkeys (Carnegie *et al.*, 2011; Charpentier *et al.*, 2018; Engh *et al.*, 2006b; Smith and French, 1997; Weingrill *et al.*, 2004; Ziegler *et al.*, 1995) and lemurs (Brockman *et al.*, 2009; Cavigelli, 1999), with birth season peaks in red-fronted brown lemurs (Ostner *et al.*, 2008) and red-bellied lemurs (Tecot, 2008), but not Verreaux’s sifaka (Fichtel *et al.*, 2007). Highest levels of GC may occur during late gestation/birth, because of glucocorticoid production in the placenta and fetus as foetal adrenal glands develop (Umezaki *et al.*, 2001), changes in circulating ovarian hormones levels and increasing metabolic demands of gestation (Setchell *et al.*, 2008). Elevated glucocorticoid levels around birth seasons also could be associated with increased protection of infants from infanticide (Ostner *et al.*, 2008). For example, when infants are nursing and most vulnerable to adult males, adult female glucocorticoid levels elevate (e.g. chacma baboons: Beehner *et al.* (2005); Engh *et al.* (2006a); mangabeys: Arlet *et al.* (2013); white-faced capuchins: Carnegie *et al.* (2011)). However, the possibility that diademed sifakas’ elevated GCs are directly caused by energetic demands of birth or the risk of infanticide seems unlikely since adult females have the most muted lean-season response (as observed in other species: Dantzer *et al.*, 2014), and the highest peaks (above 45 ng/g) were found in four immatures, one adult male and one adult female (who did not give birth that season). The most likely interpretation is that elevated GCs are primarily due to resource scarcity, and that the overlap with the birth season is coincidental.

Specifically, the sifakas’ lean-season births are thought to have evolved to allow weaning to coincide with peak resource abundance (Tecot, 2010; Wright, 1999). Roughly, conception in Tsinjoarivo sifakas occurs in December, birth occurs in June/July and weaning is completed the following February. Many females reproduce in consecutive years, and average output is roughly two births every three years. Females that birth in consecutive years experience only a short overlap between lactation and gestation (roughly January–February), thanks to high resource abundance at weaning; it seems plausible that, if weaning were timed at a less resource-rich time of year, weaning would be delayed, creating a longer period of overlap with gestation, and increased energetic challenge. Thus, the sacrifice of having birth and early lactation at the leanest time of year seems to have been evolutionarily ‘acceptable’ in return for reduced lactation-gestation overlap, making it easier to birth in consecutive years.

### Are FRAG groups more energetically challenged?

Despite a difference in peak GC levels, no systematic difference between FRAG and CONT groups was detected, which was unexpected. FRAG home ranges are smaller, and located within more disturbed habitats (Table 1), within which the fruit species consumed by CONT groups are either absent or rare (Irwin, 2008b). FRAG groups have severely reduced nutritional intakes (Irwin *et al.*, 2015). The variation within FRAG groups is also linked to habitat, with the lowest nutrient intakes seen in the groups with lowest basal area per hectare and home range quality (Irwin and Raharison, in press).

It appears the models failed to retain a Site*Season interaction because most lean-season cycles (1, 8 and 10) showed no or little elevation in GCs. We cannot rule out the possibility that the way we defined seasons was less sensitive to sifaka physiology than other methods (e.g. isolating food availability, temperature and rainfall) might have been. Lean seasons may only intermittently pose resource stress, when food availability drops below a critical level, as reflected in the clear difference between CONT and FRAG habitats in data collection cycle 9 (middle of the lean season). The FRAG groups’ muted response in cycle 9 may represent less challenging conditions (higher food abundance), or a reduced health and body condition preventing HPA activation. We consider the first explanation unlikely, because FRAG groups spend more time feeding in the lean season, yet only achieve similar or lower energy and nutrient intakes relative to CONT groups (Irwin *et al.*, 2015). It seems more likely that reduced nutrient and energy inputs throughout the year have required FRAG animals to shift their strategy. Although nutrient intake is lower in FRAG groups, it is always low; abundant and lean seasons are similar (Irwin *et al.*, 2015). With chronic nutritional challenges, FRAG groups may adopt a strategy whereby the HPA axis is stimulated only in the face of extreme stressors, thus decreasing energy expenditure by attenuating GC secretion (Fokidis *et al.*, 2012). These findings also suggest that the poorer body condition seen in FRAG sifakas (Irwin *et al.*, submitted) is not due to chronic, catabolic actions of elevated GCs.

Interestingly, the abundant season is when CONT and FRAG groups’ nutritional intakes diverge the most (Irwin *et al.*, 2015), yet the GCs diverge the most in the lean season. This suggests that FRAG groups’ reduced HPA activation in the peak lean season is not solely due to lean-season foods (indeed, CONT and FRAG nutritional intakes at this time are similar), but because FRAG animals have less fat stored. Such fat storage may be a key strategy to cope with seasonality, not just due to lower food availability, but because lean-season foods (mostly leaves) likely have higher plant secondary metabolite content than abundant season foods (mostly fruits). Additionally, given Madagascar’s interannual environmental variability (Dewar and Richard, 2007), sifakas with greater fat storage may be better equipped to survive ‘bad years’ (Lewis and Kappeler, 2005).

Overall, these results are contrary to the ‘cort-fitness hypothesis’ (Bonier *et al.*, 2009; Dantzer *et al.*, 2014), which predicts that animals in degraded habitat exhibit both lower fitness and higher GC output. This study adds to a growing list of counter-examples, for which apparently distressed animals show lower glucocorticoids (Aronsen *et al.*, 2015; Munshi-South *et al.*, 2008; Sayre, 1996; Tecot, 2008, 2013) and glucocorticoid reactivity (Homan *et al.*, 2003), or no difference (Rakotoniaina *et al.*, 2016; Rimbach *et al.*, 2013). Our results are particularly similar to patterns seen in the frugivorous, rainforest-dwelling red-bellied lemur *E. rubriventer* (Tecot, 2013); two groups in undisturbed habitat showed higher GC levels than three groups in disturbed habitat, but the key difference was that undisturbed habitat groups mounted a lean-season response (elevated GCs, increased feeding and traveling, and decreased resting) while disturbed habitat groups’ GCs and activity were largely invariant across seasons. These results suggest that lemurs’ ‘normal’ state is a challenging lean season with corresponding GC peak, and that resource stress linked to habitat degradation levels out the peak, possibly because animals lack the energy to mount a normal response and mobilize energy stores (Busch and Hayward, 2009). However, a divergent pattern was seen in collared brown lemurs, *E. collaris*, in littoral forest fragments: both groups had similar, low abundant season GC levels, but animals in more disturbed habitat had higher lean-season peaks (Balestri *et al.*, 2014). Interestingly, in contrast to sifakas, which reduce ranging, energy expenditure and feeding effort in the lean season (Irwin, 2006), the brown lemurs seem to expand ranging and feeding activity (Campera *et al.*, 2014; Donati *et al.*, 2016). Red-bellied lemurs do as well (Tecot, 2008), but only in the undisturbed forest. Thus, although lean season increases seem common across lemurs, the added effect of habitat degradation may vary according to other factors.

### Why are lean-season responses muted in females?

Glucocorticoids are primarily metabolic hormones and are expected to be higher in individuals with higher metabolic need (Beehner and Bergman, 2017). The lower lean-season GC elevation observed in adult female sifakas mirrors female responses across several species (Dantzer *et al.*, 2014), and suggests that females (1) have lower metabolic activity than adult males, (2) are less sensitive to ecological changes or (3) are unable to mount an HPA response due to a poorer energetic state.

Though the first explanation is unlikely in females of reproductive age, the second explanation may occur through lower HPA activation in adult females via behavioural strategies that buffer them from ecological and social stressors. In terms of food stressors, diademed sifakas are female-dominant and have priority access to food (Irwin, 2006), so they may be more resilient to ecological changes, particularly in having access to higher-quality foods in the lean season. In terms of social stressors, most diademed sifaka groups have few adult social partners; seven of our eight groups contained just one adult male and one adult female. In groups with more than two adults, females may have more social support, perhaps via female kin in the group, which has been associated with lower mortality in baboons (Archie *et al.*, 2014; Silk *et al.*, 2010), and lower social stress. Two-female groups have been observed three times in Tsinjoarivo sifakas; in two cases, it was a known mother–daughter pair (with observed allocarrying of infants and grooming support), while for the third, the relationship was unknown (Irwin, unpub. data). In the closely-related Milne-Edwards’ sifaka (*P. edwardsi*), both males and females disperse from natal groups, but males disperse more often throughout their lives, and have shorter longevity (Tecot *et al.*, 2013). If the same is true in diademed sifakas, males may incur cumulative costs that intensify the seasonal challenges that both sexes experience.

The third explanation proposes that females have a weaker response to seasonal ecological challenges because their stress responses are attenuated. Minimally variable GCs can indicate that individuals are experiencing extreme stress, or that sensitive individuals have been selected out of the population (Romero, 2004). In cases of extreme stress, attenuation can reduce energy expenditure as well as the likelihood of incurring chronic, pathological stress (Romero, 2004), thereby increasing survival in harsh environments. Female lemurs may have a greater energetic burden than males, resulting in a relatively attenuated response, because of the costs of reproduction (all adult females in our study were gestating and/or lactating). Future work relating individual females’ GCs to their ongoing reproduction, behaviour and health can help test this hypothesis.

### Limitations and future directions

Although our data yielded broad patterns and associations, much remains unknown about specific mechanisms and causality. Future work adding more types of data, and designed to test specific spatial and temporal associations, could fill these gaps. First, it will be important to examine associations between GCs and more direct indices of health, such as body condition or metabolic indicators of energy balance, protein turnover or immune system activation (Harrison *et al.*, 2010; Vogel *et al.*, 2012). Second, we sampled GCs intermittently (research teams rotated among sites) and on a coarse time scale; future work sampling individuals more continuously will be better poised to tease apart the influences of food, social and reproductive stress. Third, we used a simple definition of ‘lean’ and ‘abundant’ seasons reflecting both climate and fruit availability. While this uncovered broad patterns, we feel that more sophisticated ways of measuring the resource landscape, such as more frequent phenology data coupled with landscape-level resource availability estimates, would improve the context within which we interpret GC changes, behavioural shifts and fitness consequences.

In contextualizing these results, it is also important to consider that we did not sample the most-disturbed sifaka home ranges at Tsinjoarivo, FRAG1 and FRAG3. These groups were included in previous studies (Irwin, 2008b; Irwin and Raharison, in press), and had the lowest home range quality index yet recorded (3.11 and 1.02, respectively), but ceased to exist before this study began. FRAG1 lost multiple individuals to predation (Irwin *et al.*, 2009) before the single remaining individual dispersed, and the three members of FRAG3 died for unknown reasons. We hypothesized that these habitats’ food resources were barely adequate or inadequate for sustaining sifakas (Irwin *et al.* in prep.), and we suspect that these groups’ GC levels would have differed from the patterns reported here.

### Implications for conservation

Harsh environments, for example those in which resources are made scarce through anthropogenic change, can impact animals’ ability to cope with the effects of seasonal changes in food, weather and reproduction, as well as longer-term climate change. Research to date suggests that lemurs are well-adapted to lean season declines in food supply, and mount an appropriate stress response, but the interplay between this adaptation and added anthropogenic stressors needs research attention.

Evidence regarding the health of Tsinjoarivo sifakas is mixed. Broadly, our GC results found only a brief and modest difference between CONT and FRAG groups, which mirrors signals seen in morphometrics (Irwin *et al.* submitted). As a group, FRAG adults were not significantly lighter than CONT adults, and FRAG immatures were not consistently short for their age, or skinny for their length. Only in the poorest habitats were morphometric consequences evident: groups FRAG1 and FRAG3 (both of which were lost before this study) had skinnier adults and stunted immatures. These results also mirror demographic results, with FRAG and CONT groups not differing significantly in reproductive output (Irwin, unpub. data). This is somewhat at odds with FRAG groups’ reduced nutrient intakes and lower markers of physiological health in bloodwork, including lower white blood cell counts, which may suggest lower immune system readiness (Irwin *et al.*, 2010, 2015). Overall, the evidence suggests that FRAG groups at Tsinjoarivo are not in immediate danger, but their reduced nutritional status probably leaves them vulnerable to perturbation.

More broadly, further research is needed on how GC levels reflect habitat change. In our study system, GCs only differentiated between CONT and FRAG groups during a short window in the lean season, with lower levels in FRAG groups apparently reflecting greater challenge. More research is needed into the factors affecting the direction and magnitude of the GC response in degraded habitat, particularly paying attention to species’ underlying characteristics, mechanism and degree of degradation, and forest type; it is possible that reactions are non-linear (e.g. elevated GC with mild disturbance but depressed GC with more serious disturbance). Furthermore, evidence regarding the linkages between glucocorticoids, habitat degradation and fitness outcomes is rare, but sorely needed (Beehner and Bergman, 2017; Hau and Goymann, 2015). Until a greater understanding is reached, GCs will remain an imperfect window into population health and viability.
